# Modulation of Intracellular Calcium Waves and Triggered Activities by Mitochondrial Ca Flux in Mouse Cardiomyocytes

**DOI:** 10.1371/journal.pone.0080574

**Published:** 2013-11-07

**Authors:** Zhenghang Zhao, Richard Gordan, Hairuo Wen, Nadezhda Fefelova, Wei-Jin Zang, Lai-Hua Xie

**Affiliations:** 1 Department of Pharmacology, School of Medicine, Xi’an Jiaotong University, Xi’an, China; 2 Department of Cell Biology and Molecular Medicine, Rutgers, New Jersey Medical School, Newark, New Jersey, United States of America; 3 Department of Reproductive and Genetic Toxicology, National Center for Safety Evaluation of Drugs, National Institutes for Food and Drug Control, Beijing, P.R. China; Georgia State University, United States of America

## Abstract

Recent studies have suggested that mitochondria may play important roles in the Ca^2+^ homeostasis of cardiac myocytes. However, it is still unclear if mitochondrial Ca^2+^ flux can regulate the generation of Ca^2+^ waves (CaWs) and triggered activities in cardiac myocytes. In the present study, intracellular/cytosolic Ca^2+^ (Ca_i_^2+^) was imaged in Fluo-4-AM loaded mouse ventricular myocytes. Spontaneous sarcoplasmic reticulum (SR) Ca^2+^ release and CaWs were induced in the presence of high (4 mM) external Ca^2+^ (Ca_o_^2+^). The protonophore carbonyl cyanide *p*-(trifluoromethoxy)phenylhydrazone (FCCP) reversibly raised basal Ca_i_^2+^ levels even after depletion of SR Ca^2+^ in the absence of Ca_o_^2+^ , suggesting Ca^2+^ release from mitochondria. FCCP at 0.01 - 0.1 µM partially depolarized the mitochondrial membrane potential (*Δψ*_*m*_) and increased the frequency and amplitude of CaWs in a dose-dependent manner. Simultaneous recording of cell membrane potentials showed the augmentation of delayed afterdepolarization amplitudes and frequencies, and induction of triggered action potentials. The effect of FCCP on CaWs was mimicked by antimycin A (an electron transport chain inhibitor disrupting *Δψ*_*m*_) or Ru360 (a mitochondrial Ca^2+^ uniporter inhibitor), but not by oligomycin (an ATP synthase inhibitor) or iodoacetic acid (a glycolytic inhibitor), excluding the contribution of intracellular ATP levels. The effects of FCCP on CaWs were counteracted by the mitochondrial permeability transition pore blocker cyclosporine A, or the mitochondrial Ca^2+^ uniporter activator kaempferol. Our results suggest that mitochondrial Ca^2+^ release and uptake exquisitely control the local Ca^2+^ level in the micro-domain near SR ryanodine receptors and play an important role in regulation of intracellular CaWs and arrhythmogenesis.

## Introduction

Multiple calcium ion (Ca^2+^) transporters/channels have been found in the inner membrane of mitochondria [1–3]. For example, cytosolic Ca^2+^ may be sequestered via the high-capacity mitochondrial Ca^2+^ uniporter (mCU) [4–6]. The driving force for Ca^2+^ uptake is created by the mitochondrial membrane potential (*ΔΨ*_*m*_, ~ -180 mV more negative than the cytosol), which is generated by the extrusion of protons by the electron transport chain [2,7]. Conversely, mitochondrial Ca^2+^ efflux predominantly depends on the mitochondrial Na^+^-Ca^2+^ exchanger (mNCX). Under certain pathophysiological conditions, mitochondrial depolarization/dissipation causes the opening of mitochondrial permeability transition pores (mPTPs), which also act as an efflux pathway for Ca^2+^ and other small molecules (<1.5 KDa) [8]. Although controversies still remain [9,10], it has been suggested that mitochondria play an important role in the regulation of intracellular/cytosolic Ca_i_^2+^ homeostasis in cardiomyocytes under both normal and pathological conditions [11–14].

Previous work has shown that mitochondria and the endoplasmic or sarcoplasmic reticulum (ER or SR) may be physically associated through tethering structures [15,16]. In cardiac ventricular myocytes, the Ca^2+^ diffusion distance between the RyR clusters at the junctional SR and the ends of the mitochondria is approximately 50-100 nM [17], which may create a micro-domain for local Ca^2+^ and generate functional interaction between mitochondria and the SR [18]. While some studies have shown mitochondrial Ca handling may affect the dynamics and magnitudes of Ca_i_^2+^ oscillations in other cell types [19–22], it is not well understood how the mitochondria and the SR are functionally coupled with relation to the regulation of Ca_i_^2+^ homeostasis and generation of Ca_i_^2+^ waves (CaWs) and triggered activities (TAs) in cardiac ventricular myocytes. 

In the present study, we aim to test whether mitochondrial Ca^2+^ fluxes can affect this putative micro-domain [Ca^2+^] and subsequently alter the Ca^2+^ handling behavior of the SR. In particular, the regulation of intracellular CaWs by mitochondrial Ca^2+^ flux under high intracellular Ca^2+^ conditions was investigated. We found that the protonophore carbonyl cyanide *p*-(trifluoromethoxy) phenylhydrazone (FCCP) depolarizes *Δψ*_*m*_ and subsequently causes Ca^2+^ release from mPTPs, which promotes SR spontaneous Ca^2+^ release and CaWs. The exacerbation of CaWs by mitochondrial Ca^2+^ release is able to cause triggered activities, manifesting increased arrhythmogenesis during mitochondrial dysfunction.

## Materials and Methods

All animal experimental procedures were reviewed and approved by the Institutional Animal Care and Use Committee (IACUC) at the Rutgers-New Jersey Medical School, and in accordance with the *Guide for the Care and Use of Laboratory Animals*, published by the National Institutes of Health (NIH Publication No. 85-23, Revised 1996). Mice of 2-4 months old (either sex) were used in this study. All experiments were performed at 35-37°C. 

### Cell isolation

Ventricular myocytes were enzymatically isolated from mouse hearts as previously described [23]. Briefly, the hearts were removed from mice anesthetized with pentobarbital ( 0.07 mg/g, i.v.), and were retrogradely perfused at 37°C in Langendorff fashion with nominally Ca^2+^-free Tyrode’s solution containing 0.5 mg/ml collagenase (Type II; Worthington) and 0.1 mg/ml protease (type XIV, Sigma) for 10 to 15 minutes. The enzyme solution was then washed out and the hearts were removed from the perfusion apparatus. The left ventricles were placed in petri dishes, and were then gently teased apart with forceps. Finally, the myocytes were filtered through nylon mesh (200 µm). The Ca^2+^ concentration was gradually increased to 1.0 mM, and the cells were stored at room temperature and used within 8 hours. 

### Detection of mitochondrial membrane potential, *Δψ*_*m*_

Ventricular myocytes were loaded with tetramethylrhodamine methylester (TMRM, 100 nM), a lipophilic cation, for 40 min at room temperature. TMRM fluorescence was monitored with confocal fluorescence microscopy (Ex/Em: 540/580nm) and recorded using an Ixon Charge-Coupled Device (CCD) camera (Andor Technology). FCCP-induced depolarization of *Δψ*_*m*_ was assessed by the decline in TMRM fluorescence intensity. 

### Measurement of mPTP opening with calcein

Measurement of the mPTP opening was conducted as previously described [24,25]. Isolated myocytes were co-loaded with 1 μM calcein AM and 1 mM CoCl_2_ in normal Tyrode's solution at room temperature for 30 min. Loaded myocytes were washed free of calcein (CoCl_2_ was continuously perfused) just before imagining, and excited at 484 nm. Emitted fluorescence was acquired at 520 nm every 2 min. The percentage of calcein leak, estimated from the fluorescence decrease, was used as the index of mPTP opening**.**

### Measurement of mitochondrial ROS production

Changes in mitochondrial superoxide production were monitored using MitoSOX Red (Invitrogen/Molecular Probes). Isolated cardiac myocytes were loaded with MitoSOX Red (5 μM) for 30 min at 37°C followed by washout. MitoSOX Red fluorescence (EX/EM: 485/585 nm) was monitored using a Nikon Eclipse TE200 inverted microscope and recorded using an Ixon Charge-Coupled Device camera (Andor Technology) operating at ~10 fps with a spatial resolution of 500×400 pixels. MitoSOX Red fluorescence is presented as background-subtracted *F/F*_*0*_ values. The *F/F*_*0*_ value was expressed as 0 during the periods when the cell was not exposed to the excitation light. The average values measured at 3 consecutive 200 ms-exposures were used to evaluate *F/F*_*0*_ values every 2 min. The baseline value (i.e. before perfusion of FCCP) was normalized to 1. The *F/F*_*0*_ level at 6 min after FCCP treatment was compared between different groups. 

### Estimation of intracellular ATP level ([ATP]_i_) changes

The changes of intracellular ATP levels were estimated indirectly by using a fluorescent Mg^2+^ indicator (Mag-fluo-4) [26], based on the assumption that ATP serves as the dominant buffer of Mg^2+^. Thus the free intracellular Mg^2+^ concentration ([Mg^2+^]_i_) changes reflect reciprocal changes of [ATP]_i_. Ventricular myocytes were loaded with Mag-fluo-4 AM (5 µM, Molecular Probes) for 20 min in Tyrode’s solution. Mag-fluo-4 fluorescence (EX/EM: 485/530 nm) was monitored using a Nikon Eclipse TE200 inverted microscope and recorded using an Ixon CCD camera (Andor Technology) operating at ~10 fps with a spatial resolution of 500×400 pixels. The fluorescence images (3 consecutive 200 ms-exposures) were obtained every 1 or 2 min in the absence and presence of 100 nM and 1 µM FCCP, respectively. 

### Single-cell Ca_i_^2+^ measurements

Mouse ventricular myocytes were loaded with Fluo-4-AM by incubation in Tyrode’s solution containing 5 μm Fluo-4-AM for 40 min at room temperature. Ca_i_^2+^ fluorescence (EX/EM: 485/530 nm) was monitored using a Nikon Eclipse TE200 inverted microscope and recorded using an Ixon Charge-Coupled Device camera (Andor Technology) operating at ^~^50 fps with a spatial resolution of 500×400 pixels, as described in previous studies [27,28]. The fluorescence intensity was measured as the ratio of fluorescence (*F*) over basal diastolic fluorescence (F_0_). Our previous report has characterized 3 major kinds of CaWs under Ca_i_^2+^ overload condition [29]. In the present study, we carried out our experiments in cells exhibiting slow CaWs under the control condition. CaW frequencies were quantified by counting the number of spontaneous CaWs (20% increases in F/F_*0*_) and were expressed as the number of waves/min.

### Myocyte membrane potential recording using patch clamp

Myocytes were patch-clamped using the whole-cell configuration of the perforated patch-clamp technique (240 μg/ml amphotericin B). Patch pipettes (resistance = 1-3 MΩ) were filled with internal solution containing (in mM): 110 K-aspartate, 30 KCl, 5 NaCl, 10 HEPES, 0.1 EGTA, 5 MgATP, 5 Na_2_-phosphocreatine, 0.05 cAMP (pH was adjusted to 7.2 with KOH). The cells were superfused with Tyrode’s solution containing (in mM): 136 NaCl, 4.0 KCl, 0.33 Na_2_PO_4_, 4.0 CaCl_2_, 1 MgCl_2_, 10 glucose and 10 HEPES (pH was adjusted to 7.4 with NaOH). Cell membrane potential signals were measured using a MultiClamp 700A patch-clamp amplifier (Molecular Devices, Sunnyvale, Ca), controlled by a personal computer using a Digidata 1332 acquisition board driven by pCLAMP 10 software (Molecular Devices, Sunnyvale, CA). Acquisitions of cell membrane potential were carried out under the gap-free mode.

### Chemicals

Chemicals and reagents were purchased from Sigma-Aldrich (St. Louis, Mo) unless otherwise indicated. FCCP, , IAA, CsA, Ru360, oligomycin, and kaempferol were firstly dissolved in DMSO as stock solutions before diluting into the bath solutions at the final concentrations. Antimycin A stock solution was dissolved in 100% ethanol. The maximum DMSO or ethanol concentration was < 0.2% (vol/vol). 

### Statistics

Data are presented as mean ± SEM. Differences were tested for statistical significance by using, where appropriate, paired or unpaired Student’s *t*-tests, with *p* < 0.05 considered significant. In each group, the cell number (n) was obtained from a minimum of two animals.

## Results

### Effect of FCCP on Δψ_m_ and mPTP opening

**Figure 1:**
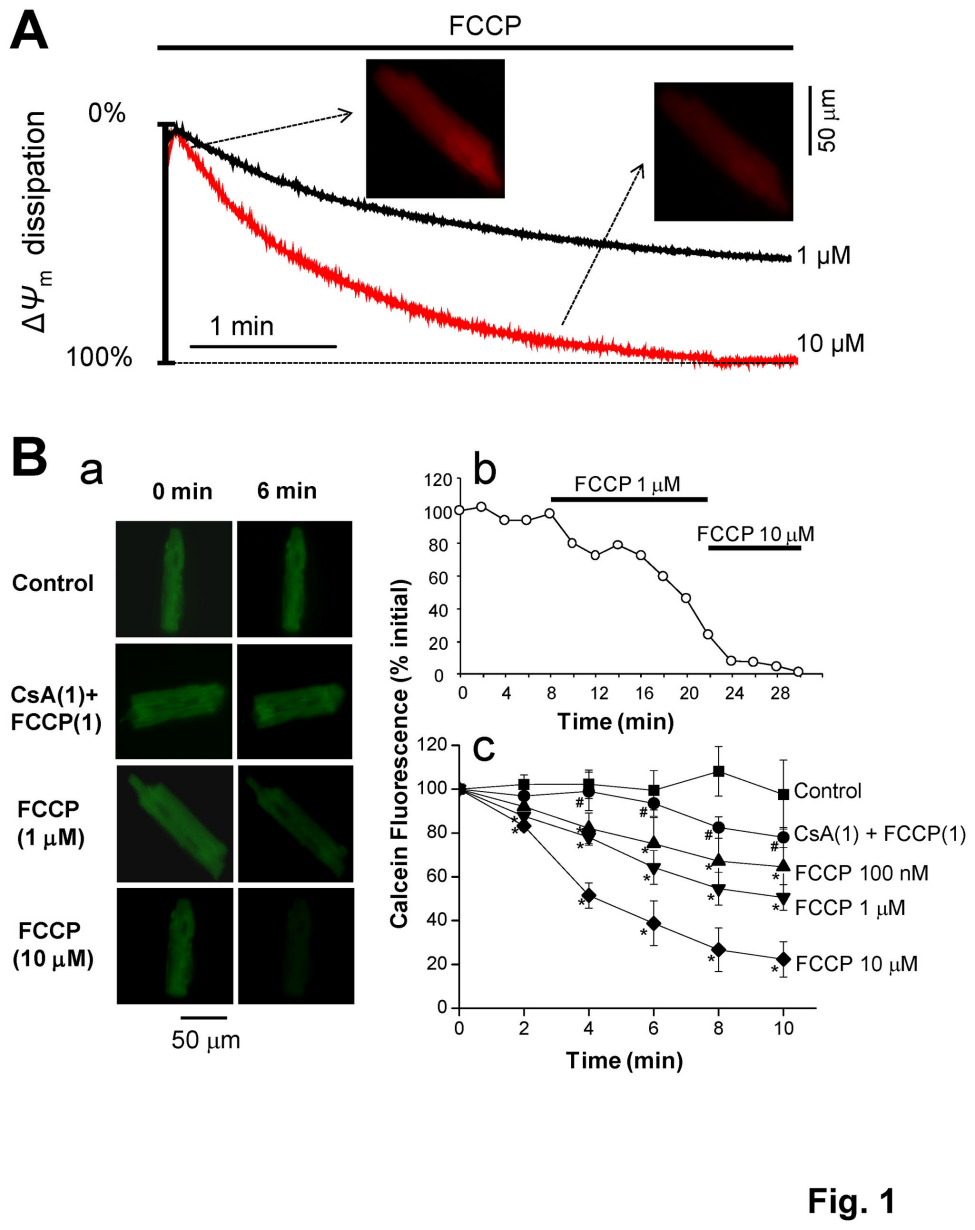
Effect of FCCP on mitochondrial membrane potential (Δ*Ψ*_m_) and mPTP opening in isolated mouse ventricular myocytes. **A**. TMRM fluorescence was monitored as an indicator of *Δψ*_*m*_. FCCP (1 and 10 µM) was perfused as indicated by the horizontal bar. Two snapshots of TMRM fluorescence (control and ~2.5 min after the treatment with 10 µM FCCP) are shown. The fluorescence in the presence of 30 µM FCCP was set as 100% dissipation in each cell. **B**. Calcein fluorescence intensity was monitored as an indicator of mPTP opening. **B**-**a**. Two snapshots of calcein fluorescence at baseline (0 min) and 6 min after the treatment with FCCP (1 and 10 µM) and FCCP (1 µM) + CsA (1 µM). **B**-**b**. A representative recording of calcein fluorescence showing the rate of fluorescence decline in the presence of 1 and 10 µM FCCP. **B**-**c**. Summary data showing the calcein fluorescence decline in the presence of FCCP (100 nM, 1 and 10 µM) and CsA + FCCP as indicated. The fluorescence in the presence of 30 µM FCCP was set as 0% in each cell. *p < 0.05 vs. Control; ^#^ *p* < 0.05 vs. FCCP (1 µM).

The electron transport chain creates a large negative potential (*Δψ*_*m*_: ~ -180 mV) across the mitochondrial inner membrane [2]. By monitoring TMRM fluorescence in intact mouse ventricular myocytes, we confirmed that FCCP strongly depolarized the *Δψ*_*m*_ in a dose-dependent manner. As shown in Figure 1A, 10 μM FCCP caused maximal dissipation of Δ*ψ*_m_ with a time constant τ = 68.5 sec, while 1 μM FCCP caused partial depolarization. Next, we evaluated whether the depolarization of *Δψ*_*m*_ causes the opening of mPTPs. As shown in Figure 1B, FCCP (100 nM, 1, and 10 μM) dose-dependently accelerated the rate of calcein fluorescence decline, which was then attenuated by the mPTP inhibitor CsA (1 µM). These results suggest that the protonophore FCCP depolarizes *Δψ*_*m*_ and causes opening of mPTP, which may further affect mitochondrial Ca^2+^ fluxes. 

### Effect of FCCP on High [Ca^2+^]_o_-induced CaWs

**Figure 2:**
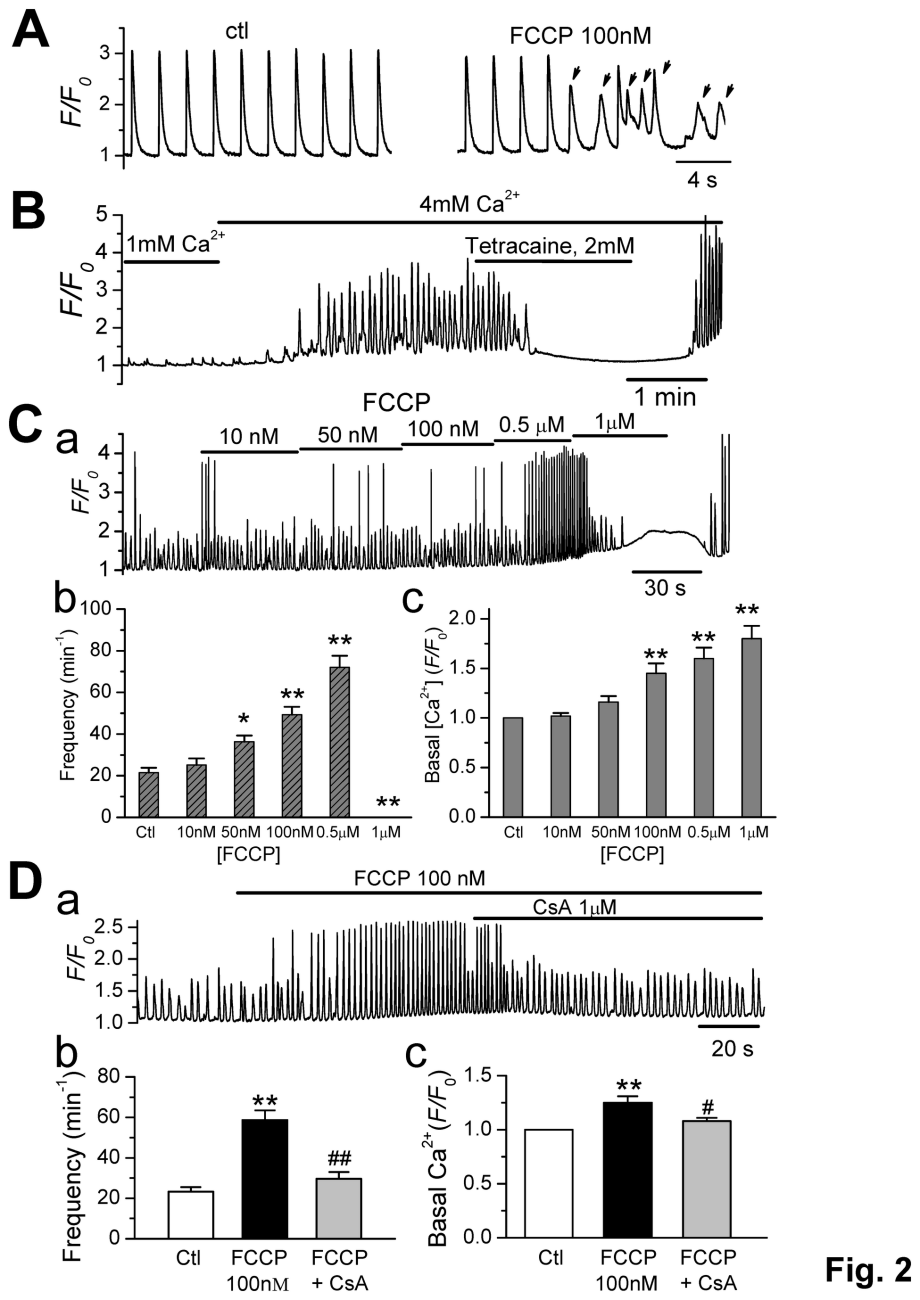
Effect of FCCP on High [Ca^2+^]_o_-induced Ca_i_^2+^ waves. **A**. FCCP (100nM) induced spontaneous CaWs (indicated by arrows) under normal excitation contraction coupling (ECC).  The cells were paced by field stimulation at 0.5 Hz in the presence of 1 mM Ca^2+^ concentration under control (ctl) condition and ~ 5 min after perfusion with 100 nM FCCP. **B**. A Ca_i_^2+^ fluorescence trace recorded from a mouse ventricular myocytes. The cell was first perfused with the normal Tyrode's solution (1 mM Ca^2+^) and then with a high Ca^2+^ Tyrode's solution (4 mM Ca^2+^). Ca_i_^2+^ waves (CaWs) were consistently observed in the presence of high external Ca^2+^ (Ca_o_^2+^; 4 mM). Spontaneous Ca^2+^ CaW were eliminated by Tetracaine (2 mM). **C**-**a**. A representative Ca_i_^2+^ fluorescence trace showing the dose-dependent effects of FCCP on the SCWs. **C**-**b**. Effect of FCCP on CaW frequency in a dose-dependent and biphasic manner. **C**-**c**. Summary data showing the effect of FCCP on basal Ca_i_^2+^. ∗*p* < 0.05,∗∗*p* < 0.01 vs. control (n = 6). **D**. Cyclosporin A (CsA), a mPTP inhibitor, significantly counteracted the effects of FCCP (100nM) on SCWs. A representative trace (**D**-**a**) and summarized data for CaW frequency (**D**-**b**) and basal [Ca_i_^2+^] level (**D**-**c**) are shown. **p < 0.01 vs. Control; ^#^ *P*<0.05, ^##^ *p* < 0.01 vs. FCCP, n = 8.

We first tested whether opening mPTP by FCCP may alter Ca_i_^2+^ handling during normal excitation contraction coupling (ECC).  The cells were field-paced at 0.5 Hz under a normal Ca^2+^ concentration (1 mM). Spontaneous CaWs and triggered Ca transients were induced by perfusion with 100 nM FCCP in 3 out of 7 cells (one example is shown in Figure 2A).

To investigate the potential regulatory effect of mitochondrial Ca^2+^ flux on SR Ca handling behaviors, we established a *consistent* CaWs model by elevating extracellular Ca^2+^ concentration ([Ca^2+^]_o_) of the Tyrode's solution to 4 mM in mouse ventricular cardiomyocytes. As shown in Figure 2B, consistent CaWs occurred 1-2 min after the perfusion solution was switched from 1 mM to 4 mM Ca^2+^. Tetracaine (2 mM), an allosteric ryanodine receptor (RyR) blocker, reversibly attenuated the CaWs. This result suggests that these CaWs originate from repetitive spontaneous Ca^2+^ release from the SR, while high [Ca^2+^]_o_ functions as a promoting factor. This high [Ca^2+^]_o_-induced CaW model was used for the subsequent experiments. 

We then examined the effect of FCCP (10 nM - 1 µM) on CaWs. A representative recording is shown in Figure 2C-a. We found that FCCP increased the frequency (Figure 2C-b) and basal intracellular Ca^2+^ level ([Ca_i_^2+^]) ( Figure 2C-c) in a dose-dependent and biphasic manner. The effects of high dose FCCP (1 µM) on CaWs (inhibition) as well as on basal Ca (elevation) were washable. Furthermore, the effects of FCCP on CaWs and basal [Ca_i_^2+^] were counteracted by the mPTP blocker cyclosporine A (CsA) (Figure 2D). The frequency of CaWs was accelerated from 23.3 ± 2.1 (control) to 57.6 ± 4.28 min^-1^ by 100 nM FCCP (*p* < 0.01, n=8), and was subsequently reduced to 27.9 ± 3.06 min^-1^ by 1 µM CsA (*p* < 0.01, n=8) (Figure 2 D-b). The basal [Ca_i_^2+^] levels were elevated from F/F_0_ =1 to 1.26 ± 0.07 by 100 nM FCCP (*p* < 0.01), and were subsequently reversed by 1 µM CsA (F/F_0_:1.05 ± 0.02*; p* < 0.05, n=8) (Figure 2D-c). In order to exclude any off-target effects of CsA, we tested the effect of CsA by itself on the Ca waves under high [Ca^2+^]_o_. CsA alone did not affect CaW frequency (control: 16.4 ± 2.5 vs. CsA (1 µM): 17.3 ± 3.1 min^-1^ (n = 5, p > 0.05). These results suggest that depolarization of Δ*ψ*_m_ (by FCCP) may affect CaW behavior via mitochondrial Ca^2+^ release through mPTP opening. 

**Figure 3:**
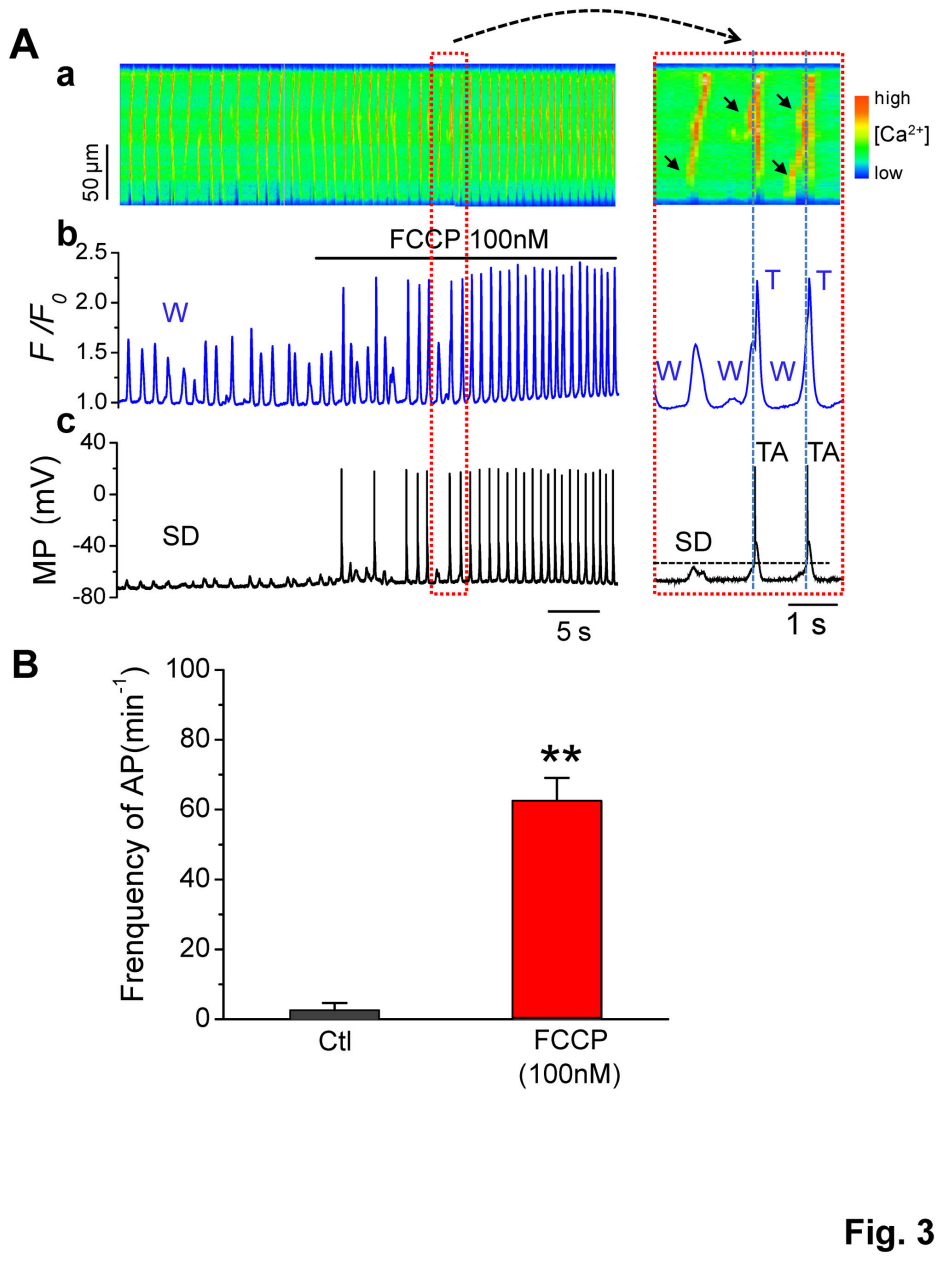
Action potentials (APs) were triggered by FCCP-enhanced Ca_i_^2+^ waves. **A**. A line-scan image along the long axis of the cell (A-a), whole-cell Ca fluorescence intensity (A-b), and membrane potential (A-c), were recorded simultaneously from a ventricular myocyte. CaWs (W) and sub-theshold depolarizations (SD) were exacerbated by FCCP (100 nM) so that triggered APs/activities (TA) and Ca transients (T) were induced. **B**. Summary data showing that FCCP (100 nM) markedly increased the incidence of TAs (**p < 0.01 vs. control, n = 4).

During perfusion with 100 and 500 nM FCCP (Figure 2C-a & D-a), high-amplitude, spike-like Ca^2+^ transients were caused, secondarily, by triggered action potentials. This phenomenon is well demonstrated in Figure 3, whereby a line-scan image of Ca^2+^ fluorescence (Figure 3A-a), whole-cell Ca^2+^ fluorescence intensity (Figure 3A-b, *F/F*_*0*_), and membrane potential (Figure 3A-c, MP) were displayed simultaneously. Only slow CaWs and correlating sub-threshold depolarizations (SDs) were observed under control conditions. After the cell was perfused with 100 nM FCCP, the amplitudes of CaW-induced SDs were increased. Triggered action potentials (TA) were induced when the SDs reached the threshold level (indicted by the horizontal dashed line in the expanded inset to the right of panel A). Conversely, TAs stimulated synchronized Ca release, which appeared as spike-like Ca transients (indicated by the vertical dashed lines). Notably, the frequency of CaW-triggered APs was profoundly raised from 3.0 ± 2.1 to 62.4 ± 6.2 min^−1^ (Figure 3B, *p* < 0.01, n = 4). Since TAs are a major mechanism for arrhythmias, these results strongly imply that mitochondria play a role in arrhythmogenesis via regulation of CaWs. The exacerbation of CaWs by mitochondrial Ca release is sufficient for triggering action potentials and thus increases arrhythmogenicity.

**Figure 4:**
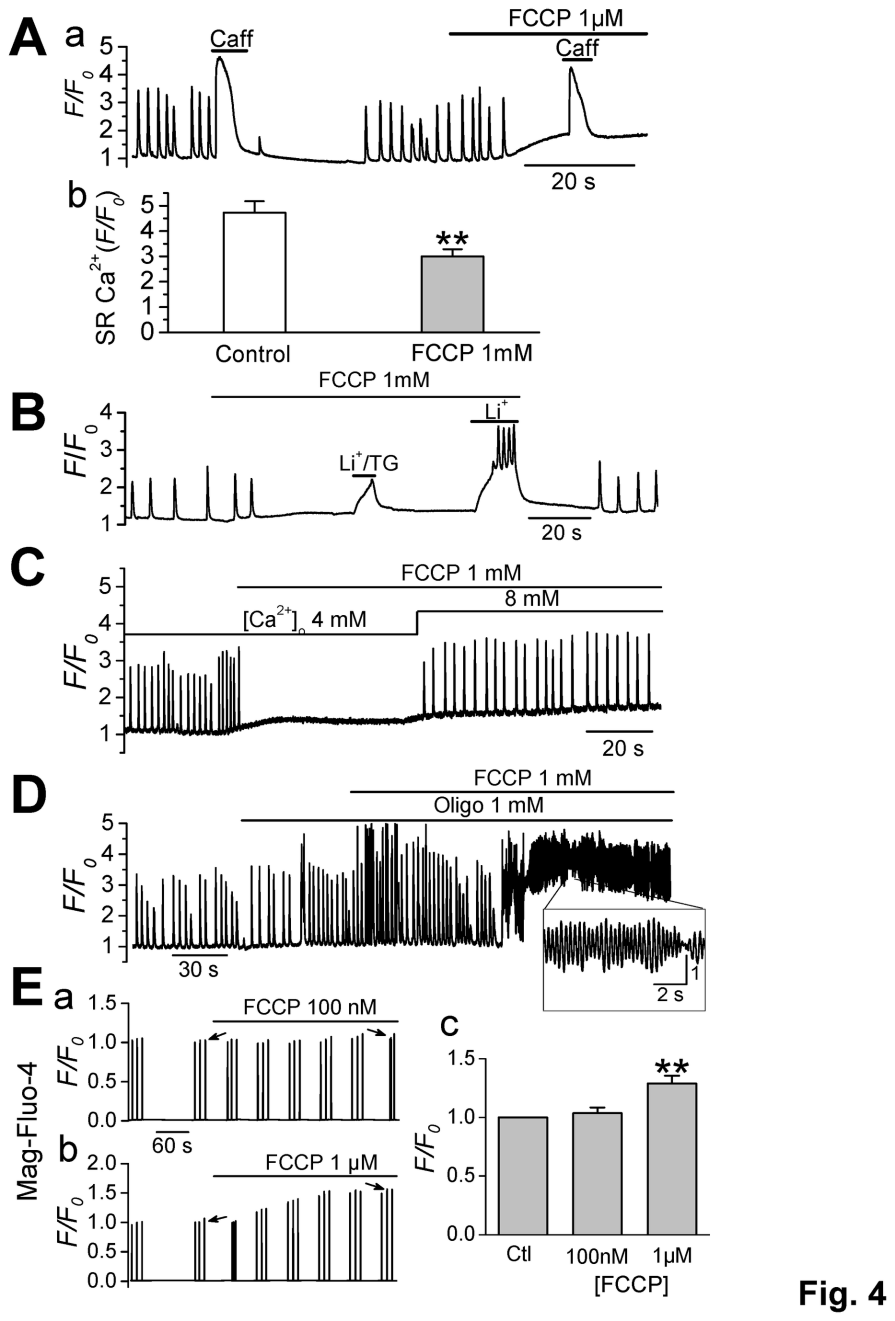
Elimination of SCWs by high dose FCCP is due to the reduction of SR Ca_i_^2+^ level via Ca^2+^ extrusion by sNCX and less SR Ca^2+^ uptake by SERCA. **A**. SR Ca^2+^ content was assessed by rapid application of 10 mM caffeine in the absence and presence of 1µM FCCP. FCCP significantly reduced SR Ca^2+^ concentration, presumably via Ca^2+^ efflux by Na^+^-Ca^2+^ exchanger (NCX) (**p < 0.01 vs. control, n = 5). **B**. Suppressing NCX restored SCWs in the myocytes pretreated with 1µM FCCP, while no SCWs were observed when SERCA2a was inhibited with thapsigargin (TG, 1µM). **C**. High-dose FCCP-inhibited CaWs were restored by elevating [Ca^2+^]_o_ from 4 mM to 8 mM. **D**. Lack of CaW inhibition by high-dose FCCP (1 µM) in the presence of Oligomycin (Oligo 1 µM). The expanded trace in the inset shows a fast Ca_i_^2+^ oscillation status. **E**. Mag-fluo-4 fluorescence (evaluation of reciprocal changes of [ATP]_i_) traces recorded in mouse ventricular myocytes treated with (**E**-**a**) 100 nM FCCP or (**E**-**b**) 1 µM FCCP. The baseline value (i.e. before perfusion of FCCP) was normalized to 1, as indicated by the leftward-facing arrow in each panel. The *F/F*_*0*_ level at 6 min after FCCP treatment (as indicated by the rightward-facing arrow) was compared between different groups. **E**-**c**. Summary data showing the intensity of the fluorescence (F/F_*0*_) recorded 6 min after the treatment (**p < 0.01 vs. Ctl).

It should be noted that FCCP at high concentrations (e.g. 1 µM) completely eliminated spontaneous CaWs, while the basal Ca^2+^ level was dramatically elevated (from F/F_0_ = 1 to 1.74 ± 0.12; *p* < 0.01, n = 8, Figure 2C). We determined that the abolition of CaWs by high dose FCCP is attributed to the reduction of SR Ca^2+^ levels via Ca^2+^ extrusion by sarcolemmal NCX (sNCX). As shown in Figure 4A, the SR Ca^2+^ content (as evaluated by a 10 mM caffeine-induced Ca^2+^ transient) was significantly decreased in the presence of 1 µM FCCP compared to the control. Blocking sNCX by a rapid application of lithium (Li^+^) raised basal Ca_i_^2+^ levels extremely high and restored CaWs (Figure 4B), while CaWs could not be restored by Li^+^ when SERCA2a was blocked simultaneously with 1µM thapsigargin. Likewise, CaWs were also restored by replenishing [Ca2+]_i_ via the elevation of [Ca2+]_o_ from 4 mM to 8 mM (Figure 4C). 

It has been shown, that in the presence of high concentration of FCCP, a protonophoric uncoupler, there is no driving force for F_*1*_F_*0*_-ATP synthase, and ATP may be consumed as the ATP synthase operates in the reverse mode. Therefore, one can assume that the decrease of SR Ca^2+^ content may be caused by the reduction of SERCA2a activity due to the lack of ATP. To test this assumption, we used oligomycin, an inhibitor of F_*1*_F_*0*_-ATP synthase, to attenuate ATP depletion in the present of FCCP. As shown in Figure 4D, 1 µM alone failed to inhibit CaWs in the presence of oligomycin (1 µM). Instead, the CaW frequency was further enhanced, with the cell entering a fast oscillation status after prolonged exposure to FCCP + oligomycin. These data suggest that the inhibitory effect of high dose FCCP was mediated, at least partially, by ATP hydrolysis through reverse-mode action of F*1*/F_*0*_-ATP synthase. It is likely the decrease of SR Ca^2+^ content at 1 µM FCCP (as shown in Figure 4A) might be due to ATP depletion and subsequent reduction of SERCA2a activity (which requires ATP). Indeed, using Mg-Fluo-4 as an indicator, we did observe the cellular ATP level was reduced (as indicated by a increase of [Mg^2+^]_i_, ) by 1 µM FCCP, but not by 100nM FCCP during the same time course (Fig. 4Ea-c).

### Effect of FCCP on mitochondrial Ca^2+^ efflux

**Figure 5:**
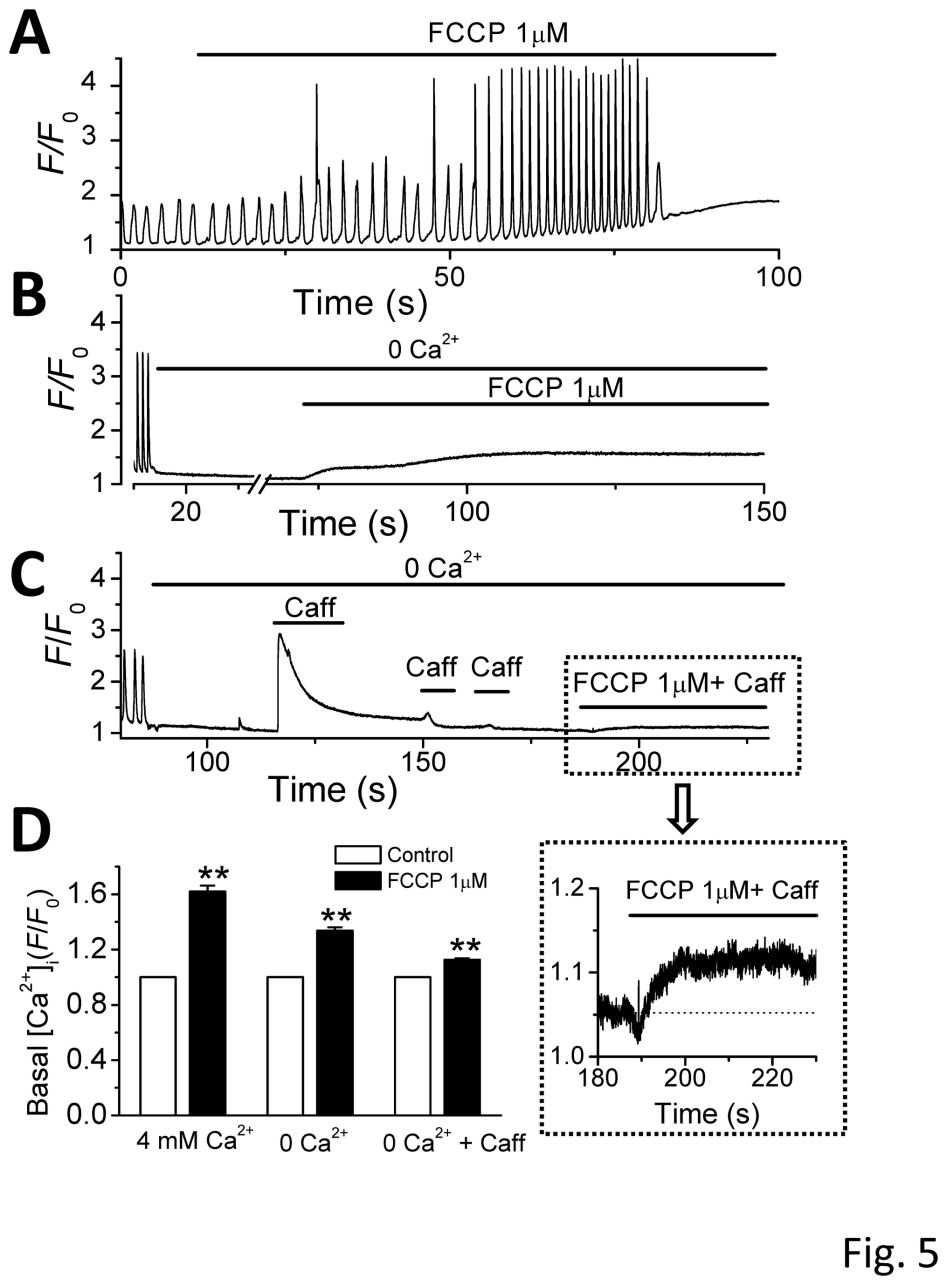
Effect of FCCP on basal [Ca_i_^2+^] level (mitochondrial Ca^2+^ efflux). **A**. Effects of FCCP (1 µM) on CaWs and basal Ca_i_^2+^ level under control condition (4 mM Ca_o_^2+^). **B**. Effects of FCCP on basal [Ca_i_^2+^] level in a Ca_o_^2+^-free condition. **C**. Effects of FCCP on basal Ca_i_^2+^ level in Ca_o_^2+^-free, and SR-Ca depleted condition. **D**. Summary data showing the effect of 1 µM FCCP on basal Ca_i_^2+^ (∗∗*p* < 0.01 vs. control, n =11).

We have observed FCCP-induced elevation of basal Ca_i_^2+^ levels (Figure 2). This elevation may represent three components: 1) mitochondrial Ca^2+^ release, 2) the entry of Ca^2+^ through the sarcolemmal membrane, and 3) SR Ca^2+^ release. In the following experiments, we dissected the component of the Ca_i_^2+^ elevation caused by mitochondrial Ca^2+^ efflux in the presence of 1 µM FCCP (Figure 5). The total basal [Ca_i_^2+^] was elevated from 1 to 1.62 ± 0.04 (n = 11; Figure 5 A and D) by 1 µM FCCP in Tyrode’s solution containing 4 mM Ca^2+^. To eliminate the component formed by the entry through the sarcolemmal membrane, we perfused the cells with a Ca^2+^-free Tyrode’s solution before adding FCCP. As shown in Figure 5B, the CaWs ceased in the Ca^2+^-free Tyrode’s solution. However, the basal [Ca_i_^2+^] was still raised by treatment with FCCP (1 μM; from 1 to 1.34 ± 0.02, n = 11), suggesting the elevated basal [Ca_i_^2+^] in the absence of extracellular Ca^2+^ can be attributed to intracellular sources, i.e. the mitochondria and SR. To further analyze the contribution from mitochondrial release, we depleted the SR Ca^2+^ by fast exposure to caffeine (10mM) before treatment with FCCP in Ca^2+^-free Tyrode’s solution. A representative recording is shown in Figure 5C. After confirming the SR Ca was completely depleted by repetitive caffeine doses, the cell was then treated with FCCP (1µM). The basal [Ca_i_^2+^] was still elevated (from 1 to 1.13 ± 0.01, n = 11) by FCCP when both extracellular and SR sources were eliminated, suggesting that mitochondrial Ca^2+^ release contributes partially to the basal [Ca_i_^2+^]. We postulate that this elevation of [Ca_i_^2+^] may locally regulate RyR Ca release function at cytosolic face of RyRs and effectively facilitate CaW and TA generation. 

### Regulation of CaWs by other mitochondrial Ca transporters

**Figure 6:**
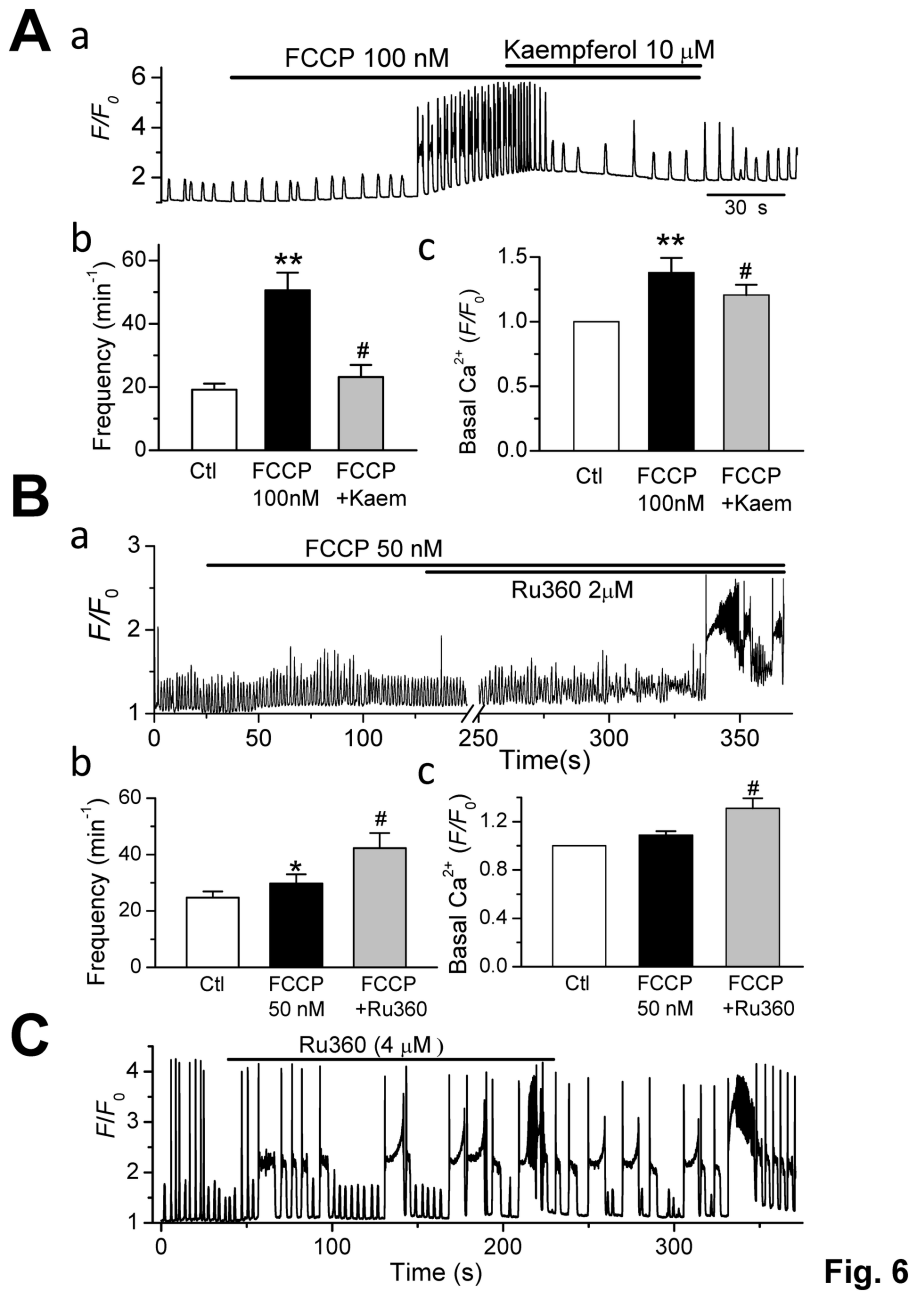
Effect of mCU Ca^2+^ flux on CaWs. **A**. Kaempferol, a mitochondrial uniporter (mCU) opener, significantly attenuated CaWs induced by 100nM FCCP. A representative trace (A-**a**) and summarized data for CaW frequency (A-**b**) and basal [Ca_i_^2+^] level (**c**) are shown (**p < 0.01 vs. control; #*p* < 0.05 vs. FCCP, n = 5). **B**. The effect of Ru360, a mCU blocker, on CaW frequency and basal [Ca_i_^2+^] level in the presence of 50 nM FCCP. A representative trace (B-**a**) and summarized data (B-b & c) are shown. ^#^ *p* < 0.05 vs. FCCP (n = 5). **C**. The effect of Ru360 on CaW in the absence of FCCP. Note a persistent and fast oscillating status after Ru360 treatment.

If local [Ca_i_^2+^] plays an important role in influencing the RyRs, and subsequently modulating CaWs, Ca^2+^ flux via other mitochondrial Ca^2+^ transporters should also be effective . This assumption was tested by modifying the function of mCUs, which mediate Ca^2+^ uptake into mitochondria [4–6]. As shown in Fig. 6Aa & b, kaempferol (10 µM), a mCU opener, significantly attenuated CaWs induced by 100 nM FCCP (*p* < 0.01 vs. FCCP, n=7). The frequency of CaWs was increased from 19.2 ± 1.9 (control) to 50.5 ± 3.9 min^-1^ by 100 nM FCCP (*p* < 0.01, n = 5), and was subsequently reduced to 23.2 ± 3.1 min^-1^ by 10 µM kaempferol (*p* < 0.05, n = 5) (Figure 6A-b). The basal [Ca_i_^2+^] level was elevated from F/F_0_ = 1 to 1.38 ± 0.09 by 100 nM FCCP (*p* < 0.01, n = 5), and was subsequently attenuated by 10 µM kaempferol (F/F_0_: 1.18 ± 0.06; *p* < 0.05, n = 5) (Figure 6A-a & c). 

In addition, we also tested the effects of the mCU blocker Ru360 on CaWs. As shown in Figure 6B, the application of 50 nM FCCP alone produced a weak effect on CaWs and basal [Ca_i_^2+^], however its effect was strongly potentiated by the addition of 2 µM Ru360. A direct potentiating effect of Ru360 on CaWs was also observed in the absence of FCCP (Figure 6C). Based on the results shown in Figure 1, we believe that only a certain percentage of total mitochondria were dissipated by 100 nM FCCP, and the voltage-dependent mCUs should still remain functional in the residual mitochondria. Thus the mCU opener keampferol or inhibitor Ru360 can be still effective in controlling mitochondrial Ca^2+^ uptake, although we cannot completely exclude the possibility they may also have other off-target effects. However, we did not observe any effect of CGP37157 (a mNCX blocker, 10 µM) on CaWs (data not shown).

### Less direct effect of metabolic inhibitors on CaWs

**Figure 7:**
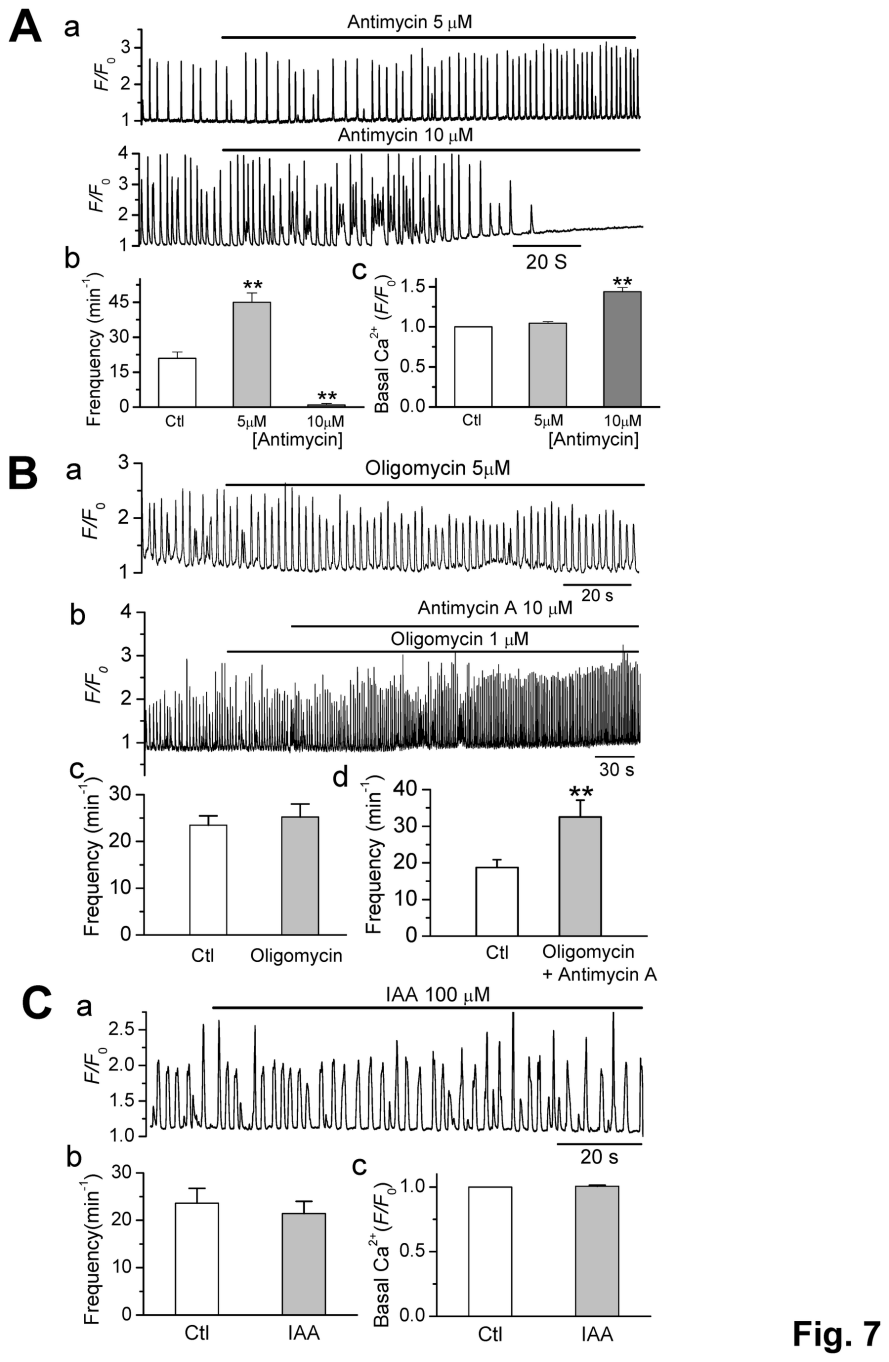
Metabolic inhibition exhibited less direct effect on CaWs. **A**. The effect of antimycin A (an electron transport chain inhibitor disrupting Δ*Ψ*_m_) on CaW frequency and basal [Ca_i_^2+^] level. Representative traces (5 and 10 µM antimycin A) (A-**a**) and summarized data (A-b & c) are shown. ∗∗*p* < 0.01 vs. control (n=6). **B**. The effects of oligomycin alone (B-a) and in the presence (B-b) of high-dose antimycine A (10 µM) on CaWs. B-b & c. Summary data showing the CaW frequencies under each condition. (n = 5 for each, ∗∗*p* < 0.01 vs. control). **C**. the same as A, except for Iodoacetic acid (IAA, a glycolytic inhibitor, n = 7) was tested. IAA exerted no significant effects on CaWs or basal [Ca_i_^2+^] (*p* > 0.05).

FCCP can also uncouple mitochondrial oxidative phosphorylation and reduce ATP production [30]. To determine whether the metabolic status is involved in the CaW regulation in our experimental conditions, we tested the effects other metabolic inhibitors have on CaW behaviors. The effect of FCCP on CaWs and basal [Ca_i_^2+^] were mimicked by antimycin A (10 µM), an electron transport chain (complex III) inhibitor, which also depolarizes Δ*ψ*_m_ (Figure 7A). The frequency of CaWs was increased from 20.9 ± 2.9 to 45.4 ± 3.5 min^-1^ (*p* < 0.01, n= 6, Figure 7A-b) before stopping, once the basal [Ca_i_^2+^] reached a high level (F/F_0_ from 1 to 1.44 ± 0.05, *p* <0.01, n = 6) (Figure 7A-c). 

On the contrary, neither oligomycin (Fig. 7Bb), an ATP synthase (F_0_F_1_-ATPase) inhibitor (n=5) nor iodoacetic acid (Figure 7C), a glycolytic inhibitor (n = 7), affected CaW frequency and basal [Ca_i_^2+^], excluding the direct contribution of intracellular metabolic condition (or ATP levels) in CaW activation under our present experimental conditions*.*

Similar to the result shown in Figure 4D, the inhibitory effect of high dose of antimycin A on CaWs was also attenuated by oligomycin (1 µM) (Figure 7B a & c), suggesting ATP depletion by reverse-mode action of F*1*/F_*0*_-ATP synthase may also be involved in CaW inhibition. 

### Less involvement of reactive oxygen species (ROS) in CaW facilitation by FCCP

**Figure 8:**
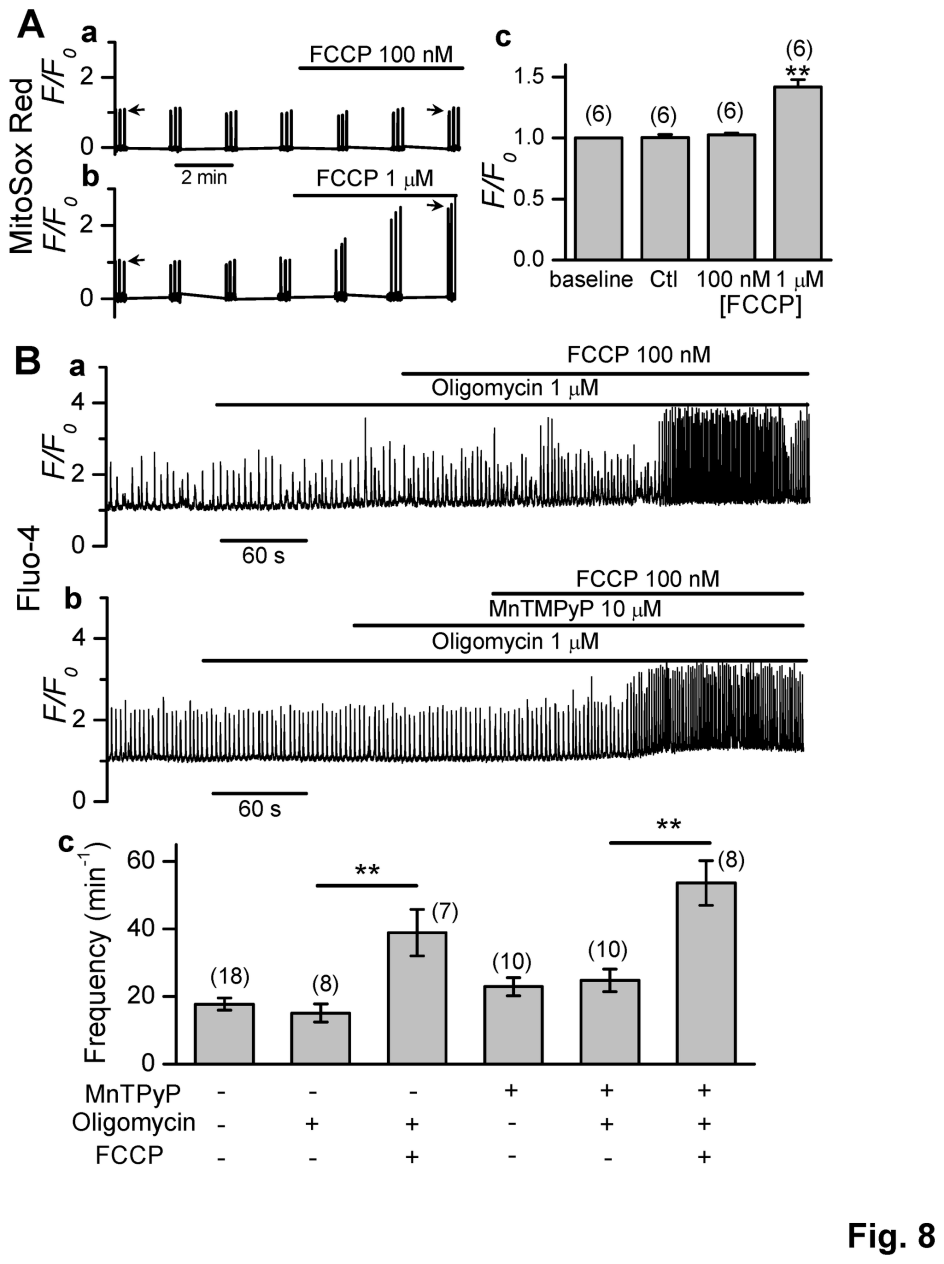
Less involvement of ROS in CaW facilitation by FCCP. **A**. Mitosox Red fluorescence (evaluation of mitochondrial superoxide level) traces recorded in mouse ventricular myocytes treated with (A-**a**) 100 nM FCCP or (A-**b**) 1 µM FCCP. The baseline value (i.e. before perfusion of FCCP) was normalized to 1, as indicated by the leftward-facing arrow in each panel. The *F/F*_*0*_ level at 6 min after FCCP treatment (as indicated by the rightward-facing arrow) was compared between different groups. (A-**c**) Summary data showing the intensity of the fluorescence (F/F_0_) recorded 6 min after the treatment (**p < 0.01 vs. Ctl). **B**. Effect of MnTMPyP (10 µM) on spontaneous CaWs. Oligomycin was applied to exclude potential influences of cellular metabolic status. FCCP (100 nM) promoted CaWs either in the absence (B-a) or the presence (B-b) of MnTMPyP. (A-**c**). Summary data showing the CaW frequencies under differential treatments. The cell number for measurement in each group is indicated (** *p* < 0.01).

It has been suggested that production of reactive oxygen species (ROS) in mitochondria may lead to aberrant calcium homeostasis [31–33]. In order to assess whether ROS was involved in the FCCP-induced regulation of CaWs in the setting of our present study, we measured mitochondrial superoxide production (by using MitoSOX Red) in the presence of FCCP at 100 nM and 1 µM, respectively. While 1 µM FCCP induced significant mitochondrial ROS generation, a lower concentration of FCCP (100 nM) did not promote mitochondrial ROS production (Figure 8A). Next, we tested the effects of antioxidants (MnTMPyP) on the frequency of spontaneous CaWs in the presence of 100 nM FCCP. Oligomycin was applied to exclude potential influences of cellular metabolic status (i.e. ATP consumption). As shown in Figure 8B, we still observed the significant activation of CaWs by FCCP in the presence of MnTMPyP, consistent with our assumption that Ca efflux via mitochondria (but neither ROS nor ATP depletion) plays a major role in the activation of CaWs by FCCP at low doses.

## Discussion

We have investigated the roles of mitochondrial Ca^2+^ flux in modulation of CaWs and TAs in mouse ventricular myocytes. The major findings of this present study include: 1) The protonophore FCCP, which can depolarize *Δψ*_*m*_ and open mPTPs, increase basal [Ca_i_^2+^] and potentiate CaWs and TAs in a dose-dependent manner in mouse ventricular myocytes; 2) the effect of FCCP on CaWs can be antagonized by CsA, an mPTP blocker; 3) Ca^2+^ flux through mCU also affects CaW behaviors; 4) the effect of FCCP on SCWs can be mimicked by antimycin A (an electron transport chain inhibitor which can also depolarize *Δψ*_*m*_), but not by oligomycin (an ATP synthase inhibitor) or iodoacetic acid (a glycolytic inhibitor). We postulate that mitochondrial Ca^2+^ flux controls the local Ca^2+^ homeostasis in the micro-domain at the cytosolic face of RyR. It is well known that the majority of RyRs are located in the dyadic regions of the junctional SR membrane and face the transverse tubule in ventricular myocytes [34]. However, at least some regions of the mitochondria (i.e. the Z-dist ends of an intermyofibrillar mitochondrion) are very close to the junctional SR and RyRs [17]. Additionally, in their recent study Lu et al revealed the presence of an intra-mitochondrial Ca gradient suggesting that there does exist the functional interaction between mitochondria and SR in terms of Ca crosstalk [18]. We postulate that mitochondrial Ca flux controls the local Ca homeostasis in the microdomain within the region close to the junctional SR and RyRs. Our study provides further evidence showing the functional coupling that occurs between the mitochondria and the SR under pathological conditions. These data implicate mitochondrial dysfunction as a potential cellular mechanism of arrhythmogenesis under stress/pathological conditions, such as ischemia-reperfusion and heart failure. A recent study by Yaniv Y, et al [35] revealed that mitochondrial, cytosolic, and SR Ca^2+^ crosstalk occurs in single rabbit sinoatrial-node cells, and regulates the automaticity of sinoatrial-node cells under physiological condition. Our current study provides insights into how a similar mechanism accounts for the arrhythmogenesis in ventricular myocytes under Ca^2+^-overload conditions. 

### Regulation of CaWs by both cytosolic and SR luminal Ca^2+^

Recent data have also demonstrated that SR Ca^2+^ leaks (Ca sparks) and CaWs are regulated by Ca^2+^ at both the SR luminal face and cytosolic face of RyRs [36,37]. Thus, elevation of cytosolic Ca^2+^ is likely to increase SR Ca^2+^ leak and facilitates CaWs by the direct activation of RyRs. Our present study provides evidence showing inter-organelle regulation between the mitochondria and SR via cytosolic Ca^2+^ and its subsequent effect on CaW regulation and generation.

We first established a CaW model by elevating [Ca_o_^2+^] (4 mM). This model indicates that Ca^2+^ influx from sarcolemma can elevate cytosolic levels and trigger CaWs. This sarcolemmal Ca^2+^ influx may be caused by non-selective cationic channels, such as TRPCs [29,38,39]. The central finding of our study is that Ca^2+^ fluxes via mitochondrial Ca^2+^ transporters dynamically change cytosolic Ca^2+^ levels and consequently regulate CaW behaviors. Although only a mild (but significant) elevation of basal [Ca_i_^2+^] induced by mitochondrial efflux was detected by measuring global Ca^2+^ (Figure 5 C and D), it is conceivable that the local [Ca_i_^2+^] at the mitochondria-SR micro-domain could be much higher.

It should be noted that FCCP has biphasic effects on CaWs (Figure 2C). FCCP promotes CaWs only at moderate concentrations (50 -500nM), while at high concentrations it suppresses CaWs (1 µM), and elevates basal [Ca_i_^2+^] (Figure 2B). Early studies on biophysical properties of RyRs reconstituted in lipid bilayers have proposed a negative feedback regulation mechanism for the regulation of RyR activities by cytosolic Ca^2+^ [40–43]. However, this negative feedback regulation cannot explain the data obtained from our experiments, since fast CaWs were restored by blocking sNCX, (substituting Li^+^ for Na ^+^ in the perfusion solution) when basal [Ca_i_^2+^] was elevated. Based on the results shown in Figure 4, we postulate that the cessation of CaWs was most likely due to the decrease of SR Ca^2+^ content below the CaW threshold level. 

We have also shown that FCCP facilitated CaWs at both low (100nM) and high concentrations (1 µM) while in the presence of oligomycin. It is known that FCCP treatment may result in ATP depletion, which is attenuated by the F_*1*_F_*0*_-ATP synthase inhibitor oligomycin. Therefore, it seems likely the biphasic effects of FCCP on CaW frequency are mediated via different mechanisms. The stimulation of CaWs by FCCP at lower concentrations could arise from increased mitochondrial Ca^2+^ release (via mPTP) and/or reduced mitochondrial Ca^2+^ uptake (via mCU) followed by increased diastolic [Ca^2+^]_i_ and enhanced SR Ca^2+^ release. On the contrary, the inhibition of CaWs at higher concentrations (e.g. 1 µM) could be caused by reduced SR Ca^2+^ content due to consumption of ATP and consequent reduction of SERCA activity, as well as excessive extrusion of Ca^2+^ through the sarcolemma. In addition, we cannot exclude the possibility of reduced RyR activity since ATP may act as a direct activator of SR RyR [44]. A recent study by Zima et al investigated how mitochondrial Ca^2+^ signaling and ATP production affect SR Ca2+ release and E-C coupling in cat atrial myocytes. They used a high dose of FCCP (2 µM) and found similar dual effects of FCCP based on Ca and ATP, respectively [26].

### Effect of FCCP on mitochondrial Ca^2+^ flux: Ca^2+^ efflux via mPTP and Ca^2+^ influx via mCU

**Figure 9:**
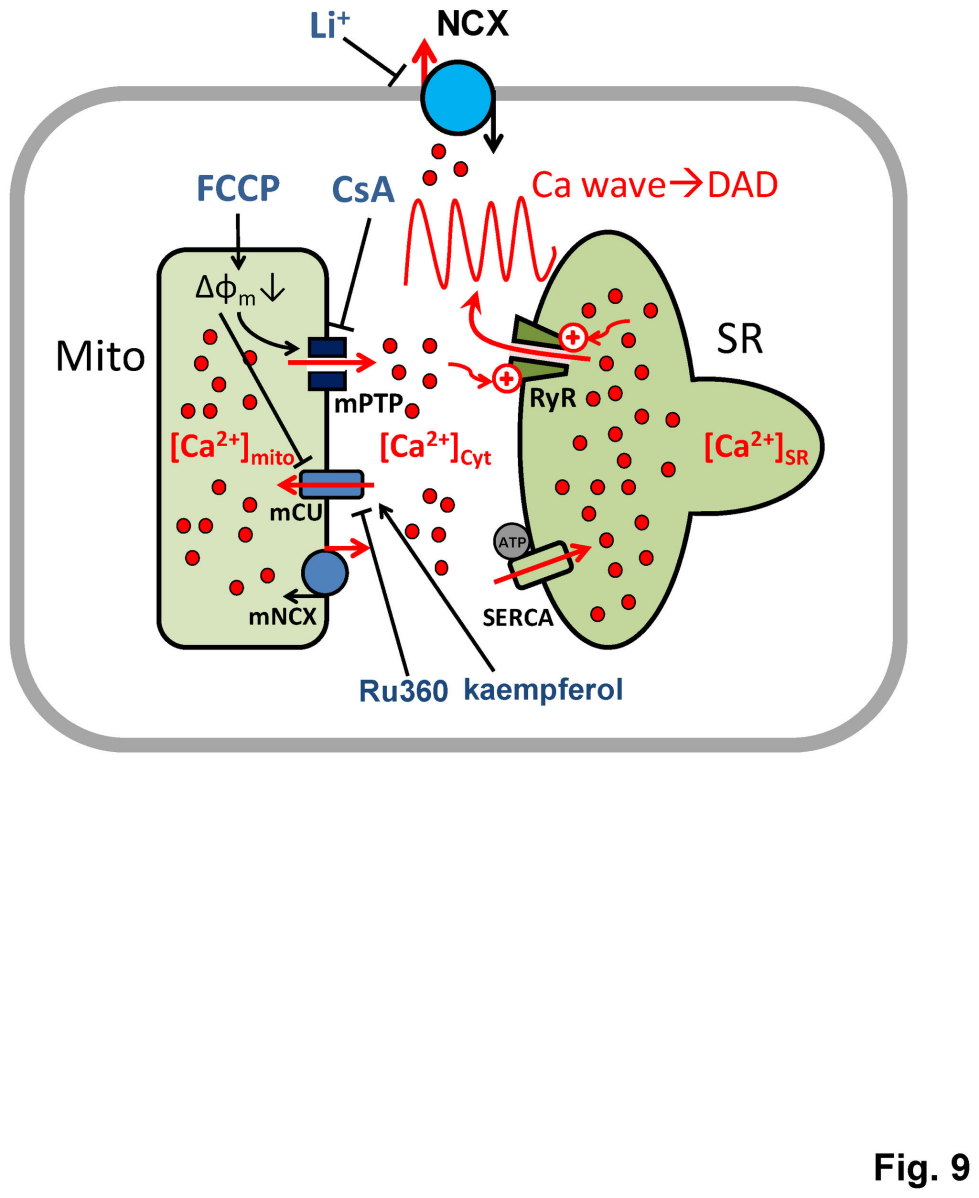
Schematic illustration of Ca^2+^ waves modulation by mitochondrial Ca^2+^ fluxes. Mitochondrial Ca^2+^ is released into the cytoplasm via the permeability transition pore (mPTP) and up-taken by the mitochondrial calcium uniporter (mCU). An increase in local cytosolic [Ca^2+^] prompts additional uptake of Ca^2+^ by the SERCA pump, and triggers a greater release of Ca^2+^ by the ryanodine receptor (RyR), thus generating/enhancing CaWs. Either inhibition of mPTP by CsA or activation of mCU by kaempferol suppresses CaWs.

As shown in the schematic illustration (Figure 9), mitochondrial Ca^2+^ flux is regulated by several mechanisms: while mCU is responsible for Ca^2+^ influx, mNCX and mPTP mediate Ca^2+^ efflux. The uptake of Ca^2+^ through mCUs is driven by the large electrochemical gradient (*ΔΨ*_*m*_ = −180 mV) across the inner mitochondrial membrane. It has been reported that FCCP depolarizes *ΔΨ*_*m*_ in RBL-2H3 cells [45], which is also confirmed in the setting of our experiments (Figure 1). Accordingly, FCCP-induced *ΔΨ*_*m*_ depolarization should reduce the driving force for Ca^2+^ uptake by mCU. Additionally, *ΔΨ*_*m*_ depolarization also leads to the opening of mPTP [4]. mPTP is a voltage-dependent, high conductance (~1200 pS) complex comprised of several components including the voltage-dependent anion channel (VDAC), the adenine nucleotide translocase (ANT), and cyclophilin D [46]. The opening of mPTPs leads to the release of mitochondrial factors less than 1.5 kDa, including Ca^2+^ and cytochrome *c* [4]. Therefore, the exacerbation of CaWs by FCCP may be mediated by reduced Ca uptake via mCU and/or increased Ca release via mPTP. This notion was supported by the results that either activation of mCU by kaempferol (Figure 6A) or inhibition of mPTP by CsA (Figure 2D) suppressed CaWs. We did not observe any apparent effect of the mNCX inhibitor CGP37157, suggesting mNCX contributes less to this CaW regulation under our experimental condition.

### Regulation of CaWs by local Ca_i_^2+^ versus metabolic inhibition

It is known that FCCP is an uncoupler of oxidative phosphorylation inhibiting the coupling between the electron transport and phosphorylation reactions and thus reducing ATP production. Since ATP is required for normal function of ionic pumps, including sarcoendoplasmic reticulum calcium ATPase (SERCA2), which mediates Ca_i_^2+^ uptake into the SR, it is possible that metabolic inhibition may cause abnormalities in Ca handling and arrhythmias. For example, inhibition of glycolysis with IAA causes elevation of diastolic Ca_i_^2+^ and a decrease of the amplitude of Ca_i_^2+^ transients in cat atrial myocytes [47]. Our previous studies have reported that inhibition of either glycolysis or oxidative phosphorylation affects Ca handling and arrhythmogenesis in embryonic mouse hearts [48]. Oligomycin (1 or 5 µM) or IAA (100 µM) by themselves did not affect CaWs, respectively (Figure 7B & 7C), suggesting that inhibition of mitochondrial ATP production (via F_*1*_F_*0*_-ATP synthase or glycolysis) *alone* was not involved in the CaW activation in the setting of our present experiments. Although we cannot exclude potential effects of long-term metabolic inhibition, our present data shows that the regulatory effects of FCCP and antimycin A are most likely due to their acute, direct effect on Ca efflux through mPTP (and reduced Ca uptake by mCU), rather than any secondary effects mediated by metabolic inhibition or ROS production. This notion is consistent with previous observations made in other cell types [22]. It should be noted that the ATP depletion induced by reverse-mode action of F*1*/F_*0*_-ATP synthase may account for, at least partially, the inhibition of CaWs in the presence of high doses of FCCP (1 µM) or antimycin A (10 µM), which are thought to cause either reduction of either SERCA2a or RyR activities, or both. 

### Pathophysiological and clinical relevance

It has been shown that mPTP opening can be induced by direct treatment with ROS [49,50] or myocardial ischemia-reperfusion (I/R) [51]. Thus, mitochondrial uncouplers, such as FCCP, have been used previously as a tool to produce cellular models of ischemia or hypoxia [52]. Our present study demonstrates that mPTP opening may cause the disruption of mitochondrial Ca^2+^ handling and therefore promote CaW generation via mitochondria-SR functional crosstalk/communication. CaWs can activate sNCX current (I_NCX_, or inward transient current I_ti_), which causes subthreshold depolarizations (SDs). The SDs trigger action potentials when they reach the threshold and predispose arrhythmias. 

 In animal models, inhibition of mPTP opening by either CsA or genetic ablation of CyP-D provides strong protection from both reperfusion injury and congestive heart failure [8,53–57], suggesting the mPTP as a promising therapeutic target in human cardiovascular disease. It has also been suggested that the mitochondria may contribute to arrhythmogenesis by introducing electrical heterogeneity into the heart tissue [58]. Inhibition of mPTP has been shown to reduce mortality following acute myocardial infarction in mice [59]. It remains unknown whether inhibition of mPTP opening may prevent arrhythmias in intact animal models or in clinical settings.

### Limitation

Our present study exclusively investigates the role of mitochondria in the regulation of cardiac myocyte function by buffering cytosolic Ca^2+^. It should be noted that mitochondrial metabolic products, such as ATP and ROS can affect SR Ca homeostasis via regulation of SERCA and RyR activity [31,60,61]. On the other hand, SR Ca^2+^ release and mitochondrial Ca^2+^ can reversely affects ATP and ROS productions through activation of the Krebs Cycle [62,63]. Thus the interaction between SR and mitochondria is complicated and can be linked via either intracellular Ca^2+^ (as shown in our present study) or intracellular metabolic products.

In order to induce CaWs consistently and study the regulatory effects of mitochondrial dysfunction, we have exclusively used a high (4 mM) [Ca^2+^]_o_-induced CaW model in this study. We believe that this model is still relevant since Ca overload may occur under various pathological conditions, such as ischemia-reperfusion, hypertrophy, and heart failure.
